# MHC class I antigen cross-presentation mediated by PapMV nanoparticles in human antigen-presenting cells is dependent on autophagy

**DOI:** 10.1371/journal.pone.0261987

**Published:** 2021-12-31

**Authors:** David Possamaï, Laïla-Aïcha Hanafi, Angélique Bellemare-Pelletier, Katia Hamelin, Paméla Thébault, Marie-Josée Hébert, Étienne Gagnon, Denis Leclerc, Réjean Lapointe

**Affiliations:** 1 Centre de recherche du Centre hospitalier de l’Université de Montréal, Montréal, Québec, Canada; 2 Institut de Recherche en Immunologie et Cancérologie, Montréal, Québec, Canada; 3 Département de Microbiologie et Immunologie, Université de Montréal, Montréal, Québec, Canada; 4 Centre de recherche en infectiologie, Centre hospitalier universitaire de Québec, Québec, Québec, Canada; 5 Département de Microbiologie, Infectiologie et Immunologie, Université Laval, Québec, Québec, Canada; 6 Département de Médecine, Faculté de Médecine, Université de Montréal, Montréal, Québec, Canada; Montana State University, UNITED STATES

## Abstract

Nanoparticles made of the coat protein of papaya mosaic virus (PapMV) and a single-strand RNA were previously shown to be an efficient antigen presentation system for the trigger of cellular immunity. Engineering of PapMV nano with a cytotoxic T lymphocyte epitope was previously shown activating specific T lymphocytes through a proteasome-independent major histocompatibility complex class I (MHC-I) cross-presentation. In this study, we provide new insights into the mechanism of the MHC-I cross-presentation mediated by PapMV nanoparticles. We demonstrate that PapMV nanoparticles do not require the transporter associated with antigen presentation (TAP), but rather depend on lysosome acidification and cathepsin S protease activity for presentation of the T cell epitope. We have also linked the induction of autophagy with this vacuolar MHC-I cross-presentation process. Interestingly, autophagy is induced in antigen-presenting cells after PapMV nanoparticles exposure and inhibition of autophagy reduce MHC-I cross-presentation. This study demonstrates that autophagy is associated with TAP- and proteasome-independent MHC-I cross-presentation. A deeper understanding of the autophagy-dependent MHC-I cross-presentation will be useful in designing vaccination platforms that aim to trigger an efficient cytotoxic T lymphocyte response.

## Introduction

Among the various vaccine vectors that have recently been developed, virus-like particles (VLPs) present multiple advantages. VLPs are protein structures composed of viral proteins assembled in a non-productive virus-like structure that do not have a viral genome. The use of VLPs reduces risks linked to productive infection by attenuated viruses while keeping their immunogenicity, making them safer vaccine candidates [1–3]. VLPs can serve as vaccine carriers through the addition of sequences coding for major histocompatibility complex class I (MHC-I) and class II epitopes from viral or tumor target proteins into the coat protein virus sequence [3, 4].

We have developed a VLP in which human leukocyte antigen (HLA)-A2 epitopes of influenza matrix protein 1 (M1) or melanoma glycoprotein 100 (gp100) was inserted at the C-terminus of the papaya mosaic virus (PapMV) coat protein sequence [5], the building block of the PapMV nanoparticles. PapMV VLPs have been studied as a promising candidate vaccine platform by displaying relevant epitopes for the induction of immune responses [6, 7]. We have previously demonstrated that the inserted epitopes are cross-presented on MHC-I by antigen-presenting cells (APC) through a proteasome-independent mechanism [8]. Also, we observed that Malva mosaic virus (MaMV) VLPs, another plant virus with a rod-like shape like PapMV VLPs, are internalized, cross-presented by APC and lead to specific T lymphocyte expansion in a similar manner than PapMV VLPs [5]. Similarly, Hepatitis B VLPs containing ovalbumin (OVA) epitope are presented on MHC class I and class II in murine CD8^+^ dendritic cells (DC) and promote cytotoxic and helper T cell priming [9]. Besides, human immunodeficiency virus-1 (HIV-1)-based VLPs coupled with E7 oncoprotein of the human papillomavirus (HPV) can cross-present E7 epitopes and induce an anti-tumor response [10]. Recently, Mohsen et al., developed a cucumber-mosaic virus-derived VLPs fused with the p33 epitope and showed an enhanced specific T cell response in the stringent B16F10p33 murine melanoma model [11]. Proteasome-independent cross-presentation has also been described for other particulate antigens, such as VLPs prepared from the rabbit hemorrhagic disease virus (RHDV), which are presented via the MHC-I recycling pathway [12]. This pathway involves processing in vacuolar compartments and depends on cathepsin S for protein degradation [13]. An antigen cross-presentation pathway dependent on recycling process and cathepsins has also been described in plasmacytoid dendritic cells (pDC), where it showed rapid potential for presentation from this particular vacuolar process [14].

In this report, we aimed to define the MHC-I cross-presentation pathway mediated by PapMV VLPs. MHC-I antigen cross-presentation pathways can be categorized into two subtypes. The cytosolic pathway where internalized antigens must access the cytosol to be processed through the classical MHC-I machinery (proteasome degradation and endoplasmic reticulum (ER) loading) [15–17]. A second pathway where degradation and loading onto MHC-I molecules are performed entirely in the vacuolar compartment [17–19]. This last pathway is defined as vacuolar or vesicular cross-presentation. Our previous data suggested that PapMV VLPs are cross-presented through a proteasome-independent pathway [8]. Therefore, we investigated the role of the transporter associated with antigen presentation (TAP), cathepsin S, and lysosome acidification in PapMV VLP cross-presentation.

Autophagy is classically described as an intracellular process that facilitates the bulk degradation of cytoplasmic materials to provide nutrients for vital cellular functions during fasting and other forms of stress. Autophagy is also implicated in the elimination of cytosolic material such as damaged organelles or protein aggregates. Some cells also use autophagy to secrete cytoplasmic constituents [20]. Moreover, it has previously been implicated in many immunological processes, including antigen presentation [20–24]. First, it plays a role in T lymphocyte development during the negative selection of CD4^+^ T cells in the thymus, where autophagy is highly implicated in MHC class II presentation of endogenous proteins [25, 26]. However, the role of autophagy in MHC-I presentation is poorly characterized. Autophagy is involved in MHC-I antigen presentation in a context where classical MHC-I presentation is inhibited by infection with the herpes simplex virus (HSV)-1 [27, 28]. Also, Budida et al., showed an increase in the capacity of murine DC to present viral antigens through MHC-I after infection with a mutant of herpes simplex virus-1 (HSV-1) which lacks infected cell protein 34.5 (ICP34.5) known to suppress autophagy, compared to the wild-type HSV-1 strain. This study demonstrates the important role of autophagy in processing endogenous viral proteins in HSV-1-infected DC [29]. Also, when TAP is inhibited, autophagy is implicated in MHC-I presentation of human cytomegalovirus [30]. Thus, we hypothesized that PapMV VLPs are cross-presented by MHC-I through an autophagy-dependent vacuolar pathway.

We report here that MHC-I cross-presentation of epitopes inserted in PapMV nanoparticles requires cathepsin S enzymatic activity and endosomal acidification. We further demonstrated that cross-presentation of PapMV VLPs was independent of TAP transport to the ER. We also identified autophagy as an important cellular activity for the MHC-I cross-presentation of cytotoxic T lymphocyte (CTL) epitopes fused to PapMV VLPs. We show that PapMV VLP exposure induces autophagy in APC. Finally, the MHC-I antigen cross-presentation mediated by PapMV VLPs requires the induction of autophagy to be efficient.

## Materials and methods

### Cells and reagents

T2 cell line was obtained from the American Type Culture Collection. EBV-B cells were generated from HLA-A2 normal donor peripheral blood mononuclear cells (PBMC) with supernatant-containing Epstein-Barr virus as previously described [31]. The protocol was approved by the ethics committees of the CRCHUM (REB: 2009–3472). All methods performed in this study were in accordance with the ethical standards of the CRCHUM and national research committees (the Fond de recherche du Québec–santé [FRQ-S], the Natural Sciences and Engineering Research Council of Canada [NSERC] and the Canadian Institutes of Health Research [CIHR]) and with the 1964 Declaration of Helsinki and its later amendments or comparable ethical standards. The participant gave written informed consent for the study. Both cell lines were cultured in RPMI 1640 supplemented with 10% (v/v) fetal bovine serum, 2 mM L-glutamine, 100 U/mL penicillin, 100 μg/mL streptomycin and 10 μg/mL gentamicin (all from Wisent Bioproducts). Rapamycin (Calbiochem) was used at 0.1–1 μM from a stock solution of 10 mM in dimethyl sulfoxide (DMSO, Sigma-Aldrich). 3-methyladenine (3-MA; cat#M9281; Sigma-Aldrich) was prepared in dimethylformamide (Sigma-Aldrich) at 100 mM stock solution. A cathepsin S inhibitor (Z-FL-COCHO, a dipeptidyl α-keto-β-aldehyde, cat#219393; Calbiochem), chloroquine (cat#C6628; Sigma-Aldrich) were prepared in DMSO at 11.7 mM and 100 mM stock solution, respectively.

### PapMV VLP preparation

PapMV-M1 and PapMV-gp100 VLPs used in this report have been described elsewhere [8].

Briefly, to generate PapMV VLPs, sense and antisense oligonucleotides coding for M1 or gp100 were annealed and cloned into at the carboxy terminus of PapMV core protein. The resulting clones, PapMV gp100 and PapMV M1, were made of the PapMV core protein gene fused with the peptide at their carboxy termini, followed by a six-histidine tag for the purification process. Five amino acids were kept on each side of the HLA A*0201 epitopes to ensure efficient processing, as in native gp100 and influenza virus M1 proteins. Sequences of the PapMV clones were confirmed by DNA sequencing.

### ShRNA lentivirus production and infection

Specific shRNAs targeting ATG5 mRNA were designed based on the sequence reference TRCN0000330394 (CCTGAACAGAATCATCCTTAA). A non-target scramble shRNA (CAACAAGATGAAGAGCACCAA) was used as a control. As described before, annealed forward and reverse hairpin oligonucleotides were cloned into a modified pLKO.1-TRC1.5 vector where the puromycin-resistance gene was replaced with mAmetrine [32]. Lentiviral particles were produced by co-transfecting HEK293T cells with shRNA-containing pLKO-mAM vector along with pMD2-VSVG, pMDLg/pRRE, and pRSV-REV, as previously described^36^. Viral supernatants were used to infect EBV-B cells. Transduced cells were sorted by flow cytometry according to mAmetrine fluorescence on a BD FACSAria III (BD Biosciences) about one week following lentivirus transduction. Validation of ATG5 knockdown was performed by western blot.

### Cross-presentation assay

EBV-B or T2 cells were pulsed with PapMV fused to M1 or gp100 epitope (PapMV VLPs; 25 μg/mL) or pulsed with M1 peptide (GILGFVFTL, 1 μM) or mutated gp100 peptide (IMDQVPFSV, 1 μM) for 6 hours. Cells were washed extensively and co-cultured overnight with antigen-specific CD8^+^ T cells. Gp100-specific CD8+ T cell clones kindly provided by Mark Dudley (National Cancer Institute; NIH, Bethesda, MD) are specific to native and modified version of gp100 HLA-A*0201-restricted epitope (209–217; ITDQVPFSV and IMDQVPFSV). T cell line specific to the influenza virus M1-derived HLA-A*0201-restricted epitope (58–67; GILGFVFTL) were generated as previously described [8]. For 4°C control, cells were kept on ice for pulsing and between washes. In assays with chemical inhibitors, cells were pretreated for 1 hour with indicated concentrations of inhibitors before adding PapMV-gp100 or gp100 peptide without washing off inhibitors and cells were pulsed for 6 hours. Co-culture supernatants were collected, and IFN-γ secreted following T cell activation was quantified by enzyme-linked immunosorbent assay (ELISA) to evaluate MHC-I antigen cross-presentation. For each experiment, the concentration of IFN-γ secreted by T cells coculture with untreated and PapMV-gp100 or gp100 peptide pulsed APC was used as reference. For assays performed with treated and pulsed APC, results are presented as the ratio of the measured IFN-γ concentration over the concentration of IFN-γ secreted by T cells coculture with untreated APC pulsed with the corresponding condition, PapMV-gp100 or gp100 peptide (% of IFN-γ secretion of untreated (UT) cells).

### Flow cytometry

PapMV VLPs were labeled with Alexa Fluor 647 fluorochrome (PapMV-AF647), according to the manufacturer’s instructions (Life Technologies). EBV-B cells were pretreated with indicated inhibitor concentrations for 1 hour before adding PapMV-AF647. Cells were then incubated for an additional 3 hours. After extensive washing, cells were analyzed by flow cytometry on a BD LSRFortessa cell analyzer (BD Biosciences). Flow cytometry data were analyzed with FlowJo Software version 10.6.1 (BD Biosciences). The gating strategy used is presented in S1 Fig.

### Western blot analysis

EBV-B cells were treated for 3 hours with PapMV nanoparticles or rapamycin, with or without pretreatment with 5 mM 3-MA. Cells were collected and washed Dulbecco’s phosphate-buffered saline (D-PBS, Wisent Bioproducts). Proteins were extracted in the presence of HALT proteinase/phosphatase inhibitors (ThermoFisher) from the above-mentioned pelleted cells and quantified by Bio-Rad protein assay. Proteins were resolved by sodium dodecyl sulfate polyacrylamide gel electrophoresis (SDS-PAGE) and transferred to polyvinylidene fluoride (PVDF) membranes (Bio-Rad). Membranes were incubated with rabbit an anti-human LC3B antibody (Ab; 1:3,000) (Novus Biologicals), a rabbit anti-ATG5-specific Ab (Cell Signaling) or a mouse anti-human β-actin-specific Ab (1:10,000) (Millipore). Membranes were revealed with horseradish peroxidase (HRP)-linked anti-rabbit (1:5,000) or anti-mouse (1:40,000 for β-actin) Abs (Chemicon) followed by enhanced chemiluminescence (ECL) prime detection (Amersham) on film. Densitometry was performed with ImageJ software and is reported as the ratio between densities of LC3-II over LC3-I bands or the ratio of ATG5 over β-actin bands.

### Confocal microscopy

EBV-B cells were transfected with a plasmid coding for LC3 fused to enhanced green fluorescent protein (eGFP), kind gift of John Brumell (University of Toronto), using a microporator (Digital Bio) with 100 μL Neon kit (Life Technologies). The next day, GFP^+^ cells were sorted by flow cytometry using a BD FACSAria III (BD Biosciences). Sorted cells were incubated with PapMV-AF647 (10 μg/mL) or rapamycin (0.1 μM) for 3 hours. Cells were cytospun using a Cytofuge 2 (StatSpin) on slides and coverslips were mounted with ProLong Gold Antifade Mountant with DAPI (4′,6-diamidino-2-phenylindole, Life Technologies). Pictures were taken on an Olympus FV100 confocal microscope and analyzed with ImageJ software for LC3-GFP^+^ vesicle count and surface quantification.

### Statistical analysis

Statistical significance (defined at p <0.05) was calculated using one sample t test. When comparing PapMV VLP cross-presentation with the cross-presentation of the control peptide at corresponding experimental condition, statistical significance (defined at p <0.05) was calculated using a two-tailed unpaired Student’s t-test; p values are indicated. When three groups were compared, statistical significance (defined at p <0.05) of normally distributed data (Shapiro-Wilk test) was calculated using a one-way ANOVA with post-hoc Tukey HSD (Honestly Significant Difference) for comparing multiple treatments or Dunnett’s multiple comparison test when means were compared to a control mean. Data were analyzed using Prism—GraphPad Software v9.2.0 (San Diego, CA, USA).

## Results

### PapMV cross-presentation is a TAP-independent active process

**Fig 1:**
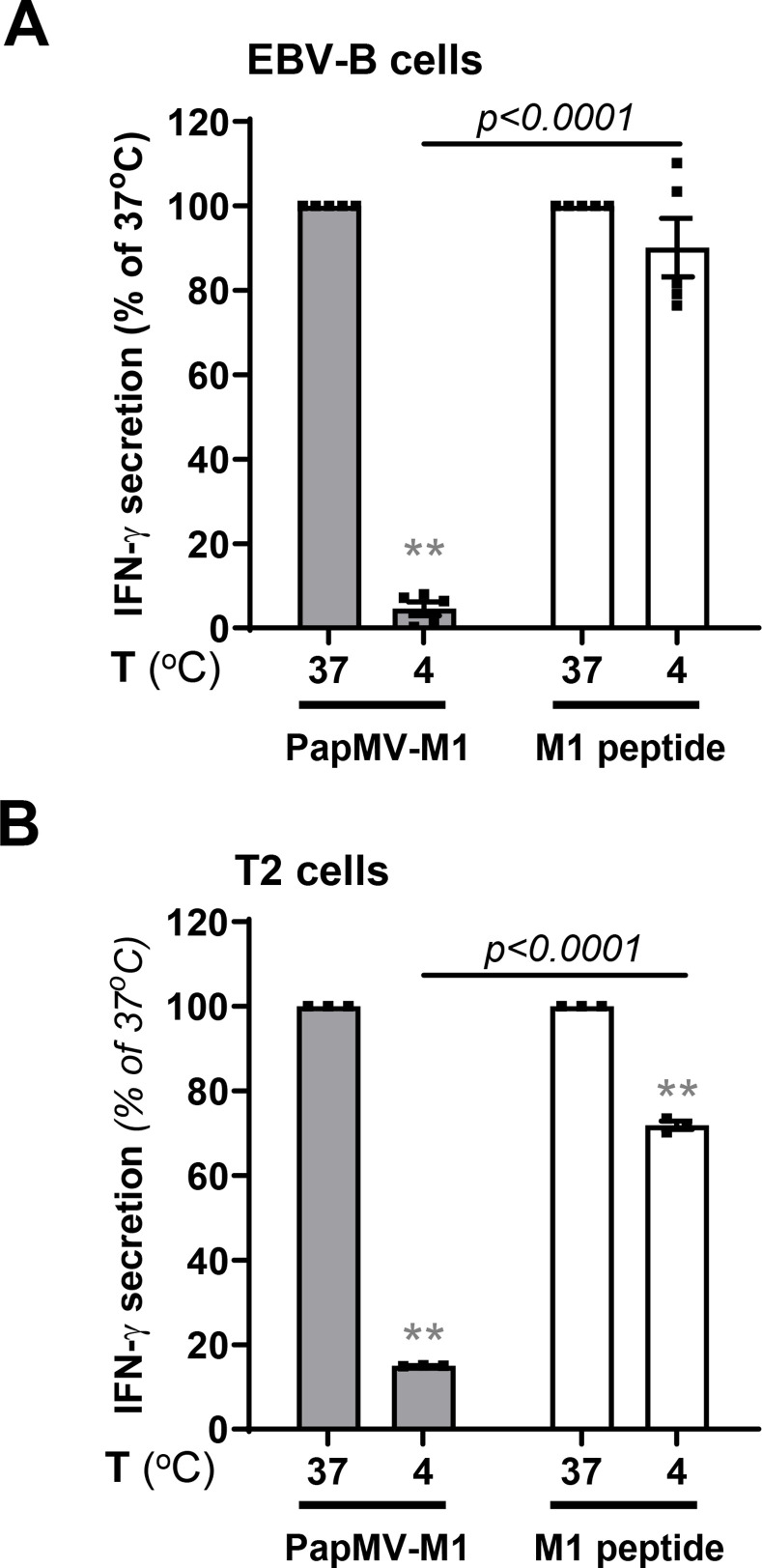
TAP-independent MHC class I cross-presentation of PapMV VLPs requires active processing. EBV-B **(A)** or T2 **(B)** cells were loaded with PapMV-M1 or M1 peptide for 6 hours at 4°C or 37°C. Cells were then washed and co-cultured with M1-specific CD8^+^ T lymphocytes for 16–18 hours at a 1:1 ratio. Supernatants were collected and IFN-γ was quantified by ELISA to evaluate MHC-I antigen cross-presentation. Results are represented as mean ± SEM (standard error of the mean), % of IFN-γ secretion at 37°C. Data were pooled from five **(A)** or three **(B)** independent experiments. Statistical significance (defined at p <0.05) was calculated using one sample t test (*p <0.05, **p <0.01). When comparing PapMV VLP cross-presentation with the cross-presentation of the control peptide at corresponding temperature, statistical significance (defined at p <0.05) was calculated using a two-tailed unpaired Student’s t-test; p values are indicated.

To study antigen cross-presentation mediated by PapMV VLPs, we developed an *in vitro* assay consisting of a 6-hour pulsing of APC with PapMV VLPs fused to specific MHC-I epitope from influenza M1 or melanoma gp100. After extensive washing, APC were co-cultured with CD8^+^ T lymphocytes specific to the inserted epitope. Assaying the activation of T cells through the secretion of interferon (IFN)-γ in the co-culture supernatant allowed us to quantify the level of cross-presentation by APC. First, we performed a cross-presentation assay in Epstein-Barr virus-transformed (EBV)-B cells incubated with PapMV-M1 at either 37°C or 4°C to verify that the release for antigen presentation of the epitope inserted in the VLP structure was mediated by an active degradation by the APC. As shown in Fig 1A, PapMV-M1 loaded APC were efficiently recognized only when pulsed at 37°C, demonstrating that degradation of the epitope fused to PapMV VLPs requires active processes. As expected, external loading of APC with M1 peptide corresponding to the minimal epitope was almost equally recognized by specific T lymphocytes when loading was performed at both 37°C and 4°C.

We performed the same antigen cross-presentation assay using pulsed T2 cells, a TAP-deficient cell line [33]. TAP is implicated in the transport of peptides from the cytoplasm to the ER. This process is essential in classical MHC-I antigen presentation and in cytosolic antigen cross-presentation [34, 35]. The efficient T lymphocyte-mediated recognition of T2 cells pulsed with PapMV-M1 suggests that antigen cross-presentation pathway is independent of TAP, and again, only cells pulsed at 37°C were efficiently recognized (Fig 1B).

These data suggest that PapMV VLP mediated cross-presentation is independent of TAP transport of the epitope into the ER and requires an active intracellular process. These results support our hypothesis that PapMV VLPs are presented by a vacuolar cross-presentation pathway.

### PapMV nanoparticles transit and are processed through the vacuolar pathway

**Fig 2:**
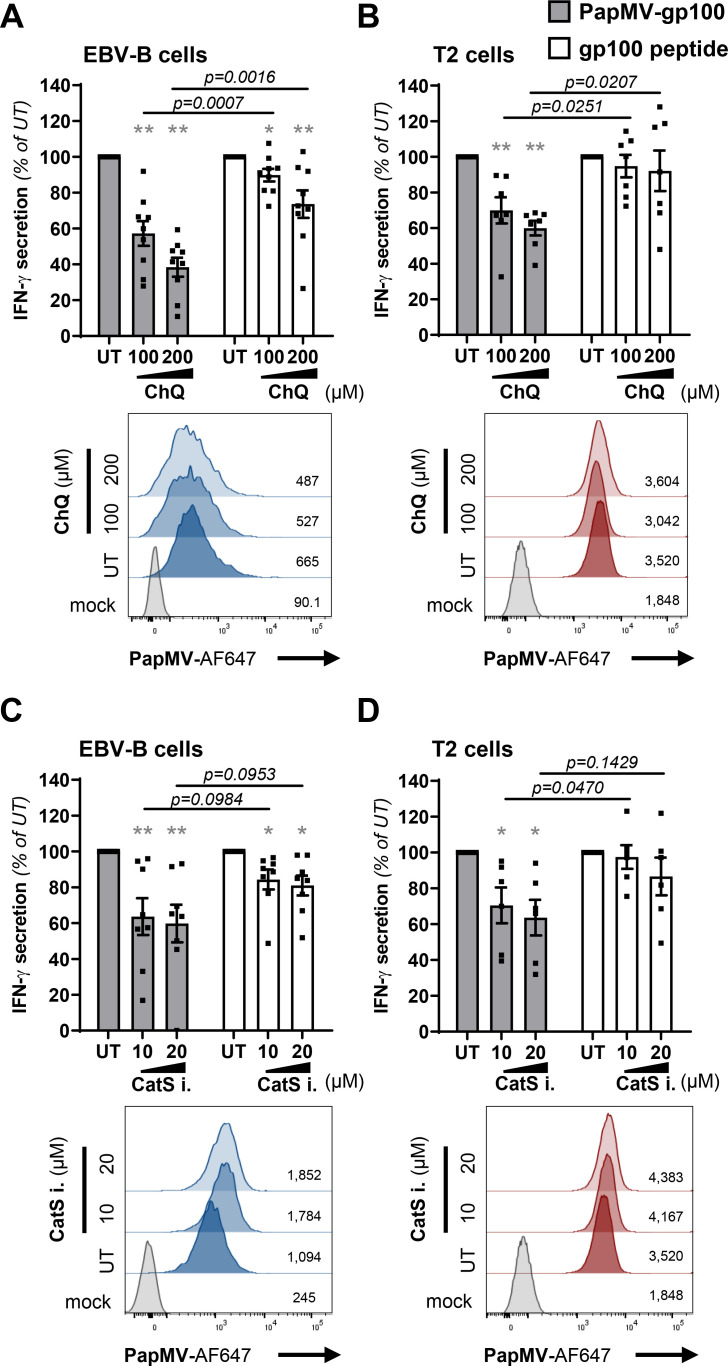
Inhibition of cathepsin S and endocytic acidification abrogate MHC class I cross-presentation mediated by PapMV VLPs. **(A-D)** Top panel: EBV-B and T2 cells were pretreated for 1 hour with chloroquine (ChQ) **(A, B)** or a cathepsin S specific inhibitor (CatS i.) **(C, D**) at indicated concentrations. PapMV-gp100 (25 μg/mL) or gp100 peptide (1 μM) was added to pretreated cells without washing and cells were incubated for 6 hours at 37°C. Cells were washed and co-cultured with gp100-specific CD8^+^ T lymphocytes for 16–18 hours at a 1:1 ratio. Supernatants were collected and IFN-γ was quantified by ELISA to evaluate MHC-I antigen cross-presentation. Results are presented as mean ± SEM, % of IFN-γ secretion of untreated (UT) cells. Data were pooled from nine **(A),** seven **(B)**, eight **(C)** or six **(D)** independent experiments. Statistical significance (defined at p <0.05) was calculated using one sample t test (*p <0.05, **p <0.01). When comparing PapMV VLP cross-presentation with the cross-presentation of the control peptide at corresponding inhibitor concentration, statistical significance (defined at p <0.05) was calculated using a two-tailed unpaired Student’s t-test; p values are indicated. **(A-D)** Bottom panel: PapMV labeled with Alexa Fluor 647 (PapMV-AF647, 10 μg/mL) was added for 3 hours and internalization was assessed by flow cytometry. Histograms represent PapMV endocytosis and are representatives of two **(A, B, D)** or three **(C)** independent experiments. Numbers indicate PapMV-AF647 MFI from the representative experiment showed.

Since our results suggested that cross-presentation of PapMV VLPs was via the vacuolar pathway, we aimed to confirm the role of previously described mediators of the vacuolar pathway (lysosomes and cathepsin S) by using chemical inhibitors. We used chloroquine, a common inhibitor that accumulates in acidic lysosomes and increases the lysosomal pH [36] thereby inhibiting lysosomal hydrolases and preventing lysosome fusion with autophagosomes. Chloroquine partially inhibited cross-presentation of PapMV-gp100, as illustrated by the reduced IFN-γ secretion by gp100-specific T lymphocytes. This reduction was significant when compared to the effect of chloroquine on the recognition of the externally loaded APCs (Fig 2A and 2B). Of note, chloroquine impacted the recognition of the externally peptide-loaded EBV-B cells but not T2 cells (Fig 2A and 2B).

Cathepsin S is a cysteine protease that has previously been linked to proteasome-independent MHC-I cross-presentation [13, 37]. When EBV-B or T2 cells were pretreated with a cathepsin S specific inhibitor before PapMV VLP pulsing, we observed a decrease in the epitope cross-presentation. We also noticed an impact of this inhibitor on the recognition of EBV-B cells, but not T2 cells, pulsed with gp100 control peptide (Fig 2C and 2D).

Using flow cytometry, we controlled whether the decrease in MHC-I cross-presentation by both inhibitors was due to cell death, a reduced internalization of PapMV VLPs or a lower expression of MHC-I molecules at the cell surface. Our results demonstrate that cell viability (S1B Fig), fluorescent PapMV VLP internalization (Fig 2C and 2D) or the expression of MHC-I molecules at the cell surface (S2C and S2D Fig) are not significantly affected by the treatment of APC with cathepsin S specific inhibitor. Similarly, treatment of EBV-B cells with chloroquine does not lead to a significant reduction of surface MHC-I molecules (S2A Fig). However, we noticed a significant decrease of the expression of MHC-I molecules at the cell surface of T2 cells treated with 100 μM of chloroquine (S2B Fig).

Collectively, our results seem to suggest that lysosomes and cathepsin S could play a role in PapMV nanoparticle processing.

### Autophagy is induced in APC by PapMV nanoparticles

Based on our results with chloroquine and our hypothesis that autophagy is implicated in MHC-I cross-presentation of PapMV VLPs, we aimed to evaluate the induction of autophagy following pulsing with PapMV nanoparticles.

**Fig 3:**
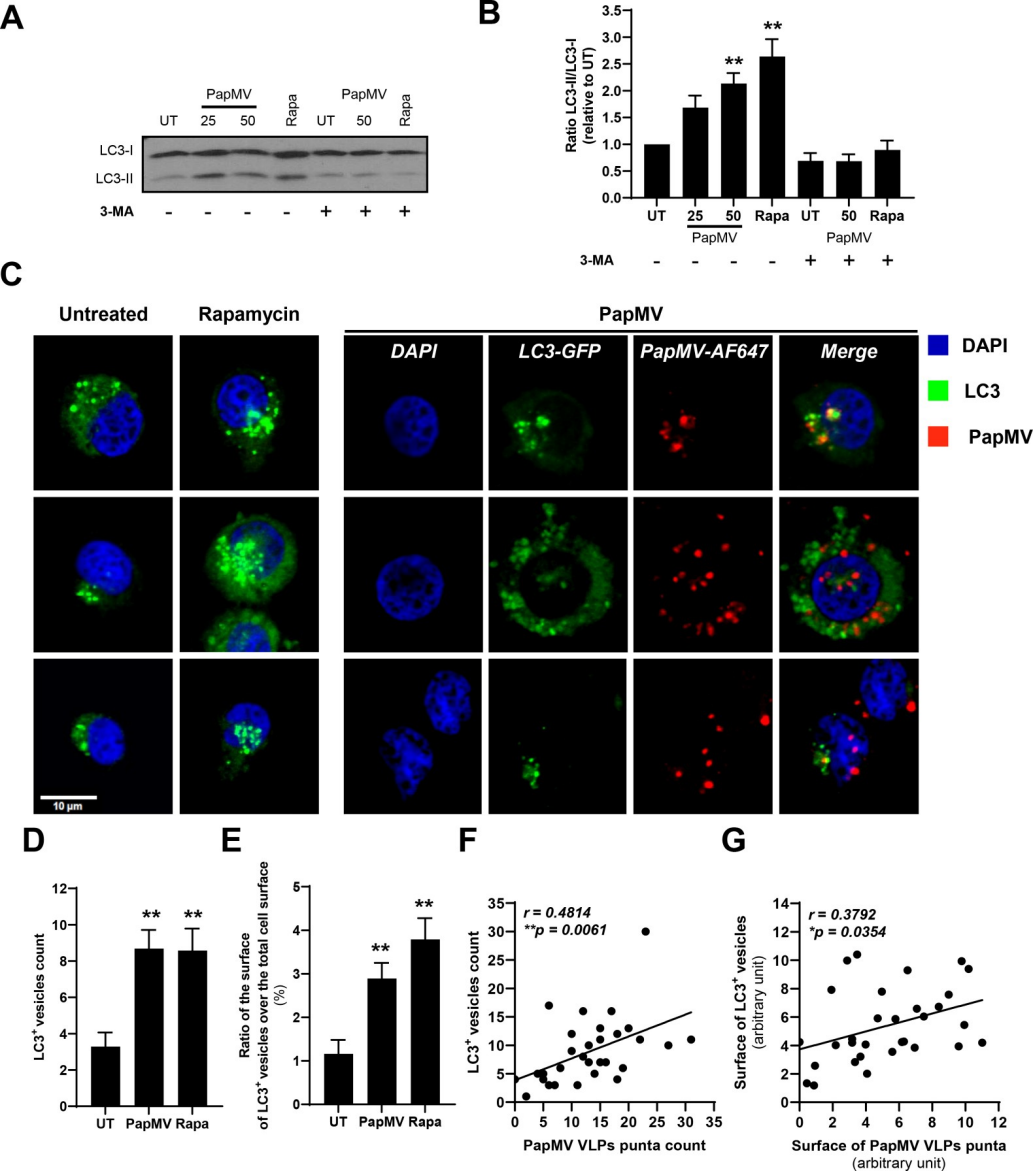
Autophagy is induced in APC following incubation with PapMV VLPs. **(A, B)** EBV-B cells were treated for 3 hours with PapMV VLPs (indicated concentration in μg/mL) or rapamycine (Rapa, 0.1 μM) with or without 1-hour pretreatment with 3-MA (5 mM). Cells were collected, protein extracted and resolved by SDS-PAGE using a 15% acrylamide gel, and LC3 was revealed by western blot. The scanned image of the blot was cropped between 20 kDa and 10 kDa for LC3 and is representative of three independent experiments. Full-length blot is presented in S5 Fig. **(B)** Ratio of densitometry of LC3-II/LC3-I bands relative to the untreated (UT) control. Data are pooled from seven (without 3-MA treatment) or three (with 3-MA treatment) independent experiments and are represented as mean ± SEM. Statistical significance was calculated using a one-way ANOVA with post-hoc Dunnett’s multiple comparison test. Statistical significance was defined at p <0.05. **p <0.01 significantly lower than UT control. **(C-E)** EBV-B cells were transfected with LC3-GFP plasmid and GFP^+^ cells were sorted and incubated for 3 hours with PapMV-AF647. **C** Pictures of representative cells per condition (untreated, PapMV or rapamycin) at 60x magnification from one representative experiment of three independent experiments. Blue: DAPI, Green: LC3-GFP and Red: PapMV-AF647. Scale bar represents 10 μm. **(D, E)** Quantification of LC3^+^ vesicles per cell **(D)** and the relative surface of LC3^+^ vesicles over total cell surface (**E**). Data are from a compilation of 24 (untreated), 32 (PapMV) and 31 (Rapamycin) cells from one representative experiment of three independent experiments and are shown as mean ± SEM. Statistical significance (defined at p <0.05) was calculated using a one-way ANOVA with post-hoc Tukey HSD. **p <0.01 significantly lower than untreated (UT) control. **(F, G)** Linear regression plots showing the correlation between **(F)** the number of LC3^+^ vesicles and the number of PapMV VLP puncta or **(G)** the surface of LC3^+^ vesicles and the surface of PapMV VLP puncta. Pearson correlation coefficients were calculated with 30 cells from one representative experiment of three independent experiments. *p <0.05 **p <0.01.

We first evaluated the induction of autophagy by western blot using the lipidated form of LC3 (LC3-II) as a marker. We showed that PapM-gp100, both at 25 or 50 μg/mL, induced the formation of LC3-II in EBV-B cells (Fig 3A). This induction can be quantified by the ratio of LC3-II over LC3-I (the cytosolic form) bands. Incubation with PapMV-gp100 at 50 μg/mL or the positive control rapamycin significantly enhanced the LC3-II/LC3-I ratio (Fig 3B). The inhibitor 3-methyladenine (3-MA) blocked the induction of autophagy by both PapMV-gp100 and rapamycin as shown by the reduction of LC3-II/LC3-I ratios (Fig 3A and 3B).

To confirm these results, we transfected EBV-B cells with a plasmid coding for a green fluorescent protein (GFP)-LC3 fusion protein to evaluate the induction of autophagy by confocal microscopy. Following transfection, GFP^+^ cells were sorted by flow cytometry and treated with PapMV VLPs labelled with Alexa Fluor 647 (PapMV-AF647), or rapamycin to induce autophagy, and imaged by confocal microscopy. As presented in Fig 3C, PapMV VLP and rapamycin exposure increased the number of LC3-GFP^+^ puncta. We quantified the formation of LC3-GFP^+^ vesicles in multiple cells and observed a 3-4-fold increase in the number of LC3^+^ puncta per cell in the presence of PapMV VLPs and rapamycin compared to untreated cells (Fig 3D). The total surface covered by GFP^+^ puncta normalized to the total cell surface was also larger in PapMV nanoparticles and rapamycin-treated cells (Fig 3E). Besides, we sometimes observed a partial colocalization between PapMV VLPs and LC3^+^ vesicles but there does not seem to be a preferred localization of PapMV-AF647 in LC3^+^ vesicles at the time point evaluated. However, the number and surface of LC3^+^ vesicles correlate with the number of PapMV-AF647 puncta and their surface, respectively, as shown by positive Pearson correlation coefficients (Fig 3F and 3G). Collectively, these results show that PapMV nanoparticles induce the formation of LC3^+^ vesicles, which seem to suggest that autophagy is induced in EBV-B cells.

### PapMV VLP cross-presentation is dependent on autophagy induction

**Fig 4:**
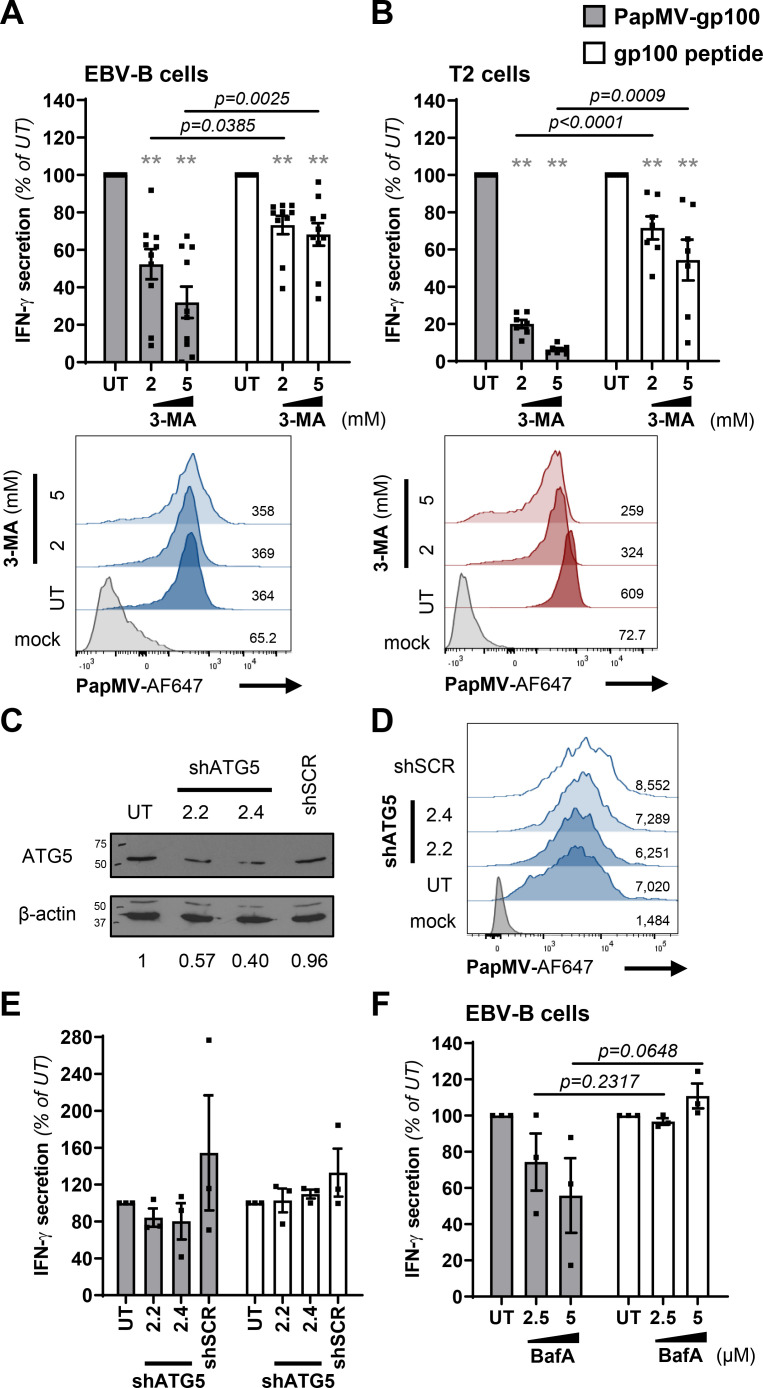
MHC class I antigen cross-presentation mediated by PapMV VLPs is dependent on autophagy induction. EBV-B and T2 cells were pretreated for 1 hour with 3-MA **(A, B)** or bafilomycin A (BafA; **F**) at indicated concentrations. PapMV-gp100 (50 μg/mL) or gp100 peptide (1 μM) were added to pretreated cells without washing and cells were incubated for 6 hours at 37°C. Cells were washed and co-cultured with gp100-specific CD8^+^ T lymphocytes for 16–18 hours at a 1:1 ratio. Supernatants were collected and IFN-γ secretion was quantified by ELISA to evaluate MHC-I antigen cross-presentation. Results are presented as mean ± SEM, % of IFN-γ secretion compared to untreated cells from ten **(A)**, seven **(B)** and three **(F)** independent experiments. **(C-E)** EBV-B cells were transduced with lentivirus expressing mAmetrine and shRNA targeting ATG5 mRNA (clones 2.2 and 2.4) or scramble shRNA (shSCR). After expansion of transduced cells, mAmetrine^+^ cells were sorted by flow cytometry. **(C)** ATG5 knockdown was evaluated by western blot. Cells were collected, protein extracted and resolved by SDS-PAGE using a 10% acrylamide gel. ATG5 and β-actin were revealed by specific antibodies on the same membrane. Scanned images of the blot were cropped between 50 kDa and 75 kDa for ATG5 and 37 kDa and 50 kDa for the β-actin loading control and are representative of two independent experiments. Full-length blots are presented in S5 Fig. Numbers represent densitometry of ATG5/β-actin relative to untransduced cells (UT) for the blot presented. **(A, B, D)** PapMV-AF647 (10 μg/mL) was added for 3 hours and PapMV-AF647 internalization was assessed by flow cytometry. Representative histograms in EBV-B **(A, D)** or T2 **(B)** cells from three independent experiments. Numbers indicate PapMV-AF647 MFI from the representative experiment showed. **(E)** PapMV-gp100 (50 μg/mL) or gp100 peptide (1 μM) was added and cells were incubated for 6 hours at 37°C and a coculture assay was performed as described above. Results are presented as mean ± SEM, % of IFN-γ secretion compared to untreated (UT) cells. Data were pooled from three independent experiments. **(A, B, E, F)** Statistical significance (defined at p <0.05) was calculated using one sample t test. Statistical significance (defined at p <0.05) was calculated using a two-tailed unpaired Student’s t-test when comparing PapMV VLP cross-presentation with the cross-presentation of the control peptide at corresponding inhibitor concentration; p values are indicated.

To investigate on the implication of autophagy in MHC-I cross-presentation of PapMV VLPs, we treated APC with 3-MA to inhibit autophagy induction during PapMV-gp100 or gp100 peptide pulsing. Pretreatment of APC with 2 mM or 5 mM 3-MA resulted in significant reductions of MHC-I cross-presentation mediated by PapMV-gp100 compared to gp100 peptide-pulsed APC after similar treatment (Fig 4A and 4B). We controlled the impact of 3-MA treatment on the uptake of fluorescent PapMV nanoparticles and the expression of MHC-I molecules. We showed that this inhibitor had no impact on PapMV-AF647 endocytosis in EBV-B cells (Fig 4A) but induced a decrease of PapMV-AF647 uptake in T2 cells (Fig 4B). We also evaluated the effect of 3-MA treatment on MHC-I expression. Treatment of APC with 3-MA had no impact on cell viability at both concentrations (S1B Fig). Similarly, treatment with 3-MA at 2 mM had no impact on MHC-I expression but a significant decrease of MHC-I MFI was observed when cells were incubated with 3-MA at 5 mM (S2 Fig). In T2 cells, 3-MA treatment significantly decreased MHC-I expression on the cell surface both at 2 mM and 5 mM as shown by a lower MFI compared to untreated cells (S2E Fig). Those results may explain the stronger effect of 3-MA on the reduction of PapMV VLP cross-presentation seen in T2 cells compared to EBV-B cells (Fig 4A and 4B). Although 3-MA has non-specific effects on APC, our data indicate that MHC-I cross-presentation of PapMV-gp100 is decreased when APC are treated with the autophagy induction inhibitor 3-MA.

We also used RNA interference as an alternative method to inhibit autophagy. EBV-B cells were transduced with lentivirus expressing mAmetrine (fluorescent protein) and short hairpin RNA (shRNA) against autophagy related 5 (ATG5) mRNA (shATG5) or a scramble shRNA (shSCR). After sorting for mAmetrine^+^ cells, we obtained a population of EBV-B cells having a 40–60% ATG5 knockdown (KD) as evaluated by western blot densitometry (Fig 4C). Internalization of PapMV VLPs was not affected by ATG5 KD by both shRNAs compared to shSCR and untransduced cells (Fig 4D). Although we observed a small downregulation of MHC-I cross-presentation in ATG5 KD EBV-B cells pulsed with PapMV-gp100 compared to peptide-pulsed EBV-B cells transduced with the same shRNA (Fig 4E), we cannot conclude on the role of ATG5 in our experimental models.

We treated EBV-B cells with bafilomycin A (BafA) to inhibit lysosome fusion with autophagosomes during PapMV-gp100 or gp100 peptide pulsing. Although non statistically non-significant, pretreatment with 2.5 μM or 5 μM BafA resulted in an apparent decrease of MHC-I cross-presentation mediated by PapMV-gp100 compared to gp100 peptide-pulsed EBV-B cells after similar treatment (Fig 4F).

Finally, we also evaluated whether the induction of autophagy before the recognition assay would increase levels of IFN-γ secretion by specific T lymphocytes. We induced autophagy by pretreating EBV-B and T2 cells with rapamycin as in Fig 3. Surprisingly, this treatment reduced antigen presentation of both PapMV-gp100 and externally loaded gp100 peptide. We controlled whether these results were due to cell death, a reduced internalization of PapMV VLPs or a lower expression of MHC-I molecules at the cell surface. We show that rapamycin treatment has no significant impact on cell viability (S1 Fig), PapMV-gp100 uptake or the expression of surface MHC-I (S3 Fig). We then assessed whether cell starvation, an inducer of autophagy through the mammalian target of rapamycin (mTOR) pathway [38] would increase PapMV-gp100 cross-presentation. We incubated EBV-B cells for 3 hours in HBSS (Hank’s balanced salt solution) during the 6-hour antigen loading and proceeded with the recognition assay as described above. Incubation in HBSS for 3 hours induced the lipidation of LC3 in EBV-B cells and a higher antigen recognition of EBV-B cells pulsed with PapMV-gp100 but not with the minimal epitope (S4 Fig). However, this result could not solely be explained by an enhanced processing of PapMV-gp100 when autophagy was induced due to a higher endocytosis of PapMV nanoparticles under starvation conditions (S4 Fig).

Overall, although our results seem to point toward the implication of autophagy in MHC-I vacuolar cross-presentation of PapMV nanoparticles, additional data are required to fully conclude on the implication of autophagy and its mechanism associated to PapMV VLPs processing.

## Discussion

MHC-I cross-presentation is an essential pathway for the priming of CD8^+^ T lymphocytes. When APC are not infected by pathogens or when cancer antigens are not expressed in APC, the induction of cross-presentation using a vaccine platform is an attractive method to improve uptake and presentation of antigens to prime naive CD8^+^ T cells and mount an effective cellular immune response.

Multiple pathways of MHC-I cross-presentation have been described. Some processes depend on the conventional pathway where endocytosed antigens are transferred into the cytosol for their degradation by the proteasome and loading of resulting epitopes on MHC-I in the ER (reviewed by Blander et al. [17]). In our system, we have previously shown that epitopes inserted in the PapMV VLP vaccine platform were presented on MHC-I through a proteasome-independent mechanism [8]. In this study, we propose that MHC-I cross-presentation of PapMV VLPs is TAP-independent but dependent on lysosomes and cathepsin S. Many viruses have evolved to inhibit TAP activity to evade antiviral immune surveillance [39–41]. However, the immune system has developed different MHC-I presentation pathways that do not require TAP activity [42, 43]. Interestingly, TAP-deficient individuals are still able to present viral associated antigens and harbor a polyclonal CD8^+^ T cell repertoire capable of recognizing peptides from the Epstein-Barr virus [44]. Thus, the TAP-independent pathway is efficient to induce CD8^+^ T cell immunity and our data support the use of PapMV VLPs as a very efficient and attractive technology for developing a vaccine platform able to generate a cellular immunity against inserted epitopes.

Autophagy has been implicated in alternative MHC-I antigen presentation pathways [23]. English et al. [27, 28], showed that in late stages of infection by HSV-1, a novel form of autophagy using the nuclear envelope is implicated in MHC-I antigen presentation. This antigen presentation pathway still required the MHC-I classical machinery, including proteasome and TAP. In contrast, when using TAP-deficient cells, Tey et al. [30], demonstrated that processing of an endogenous antigen from the human cytomegalovirus (HCMV) occurred completely in the vacuolar pathway and was mediated by macroautophagy. While previous studies required active infection and protein translation for classical MHC-I presentation, in our study, we propose that antigen cross-presentation of epitopes inserted in PapMV VLPs is mediated by the same vacuolar that seems to depend on autophagy.

We showed that inhibition of autophagy by chloroquine and 3-MA reduced the cross-presentation of PapMV VLPs. Although further data are required to fully confirm our results, our data suggest that pretreatment of EBV-B cells with bafilomycin A seem to lead to a reduction of PapMV VLP cross-presentation. Bafilomycin A inhibits the fusion of autophagosomes with lysosomes but is also an inhibitor of the Vacuolar-type ATPase (V-ATPase), therefore inhibiting the acidification of endosomes similar to chloroquine. Hence, it might be possible that the observed reduction of PapMV nanoparticles cross-presentation when APC are treated with both these inhibitors might be due to an inhibition of the activity of several lysosomal proteases implicated in PapMV nanoparticles processing and not the inhibition of autophagy per se.

Our data also show that chloroquine, cathepsin S inhibitor and 3-MA impacted the recognition of the externally peptide-loaded APC especially EBV-B cells, though in a reduced manner compared to PapPV VLP-pulsed APC. These data suggest these inhibitors have non-specific effects on both cell types which impact APC presentation ability. Although this is a matter of a separate study, further data are required to fully decipher the impact of these inhibitors on APC and PapMV VLPs processing.

To demonstrate the role of ATG5 in the cross-presentation of PapMV VLPs, we used shRNAs targeting ATG5 mRNA. However, this strategy only led to a limited ATG5 knockdown. As ATG5 functions are redundant, this knockdown was not sufficient to lead to a significant impairment of PapMV VLP cross-presentation. Of note, several other shRNAs targeting ATG5 mRNA were also evaluated to improve ATG5 knockdown. Unfortunately, most shRNA induced EBV-B cell death few days after cell sorting. These results may be explained by the important role of autophagy in EBV-B cell survival [45]. Targeting other proteins implicated in autophagy such as ATG7 using shRNAs or CRISPR-Cas9 technology as well as performing double knockdowns (e.g., AT5-ATG12 knockdowns) could provide a mean to circumvent this limitation.

Recently, Dasari et al. [46], showed that EBV-B cells can cross-present a soluble recombinant HCMV-encoded pp65 protein through a proteasome-dependent but TAP-independent cross-presentation pathway to CD8^+^ T cells. Similar to our data, cross-presentation was inhibited by pretreating B cells with chloroquine, 3-MA or by ATG5 or ATG12 knockdown, strongly supporting that cross-presentation of pp65 was mediated by autophagy. The authors proposed a new antigen cross-presentation pathway by B cells involving processing of antigens through an autophagy- and proteasome-dependent pathway and loading of peptides onto MHC-I proteins in autophagosomes. Overall, this suggests that several cross-presentation pathways may rely on autophagy for processing and presentation of different types of antigens.

We also evaluated whether the induction of autophagy before the recognition assay would increase levels of IFN-γ secretion by specific T lymphocytes. The treatment of APC with rapamycin surprisingly led to a reduced antigen presentation of both PapMV-gp100 and externally loaded gp100 peptide which could not be explained by a decrease in VLP uptake or a downregulation of surface MHC-I by rapamycin treatment. Further experiments are needed to decipher the impact of rapamycin on antigen presentation by EBV-B and T2 cells. We also assessed whether cell starvation would increase PapMV-gp100 cross-presentation. Starvation induced autophagy in EBV-B cells and lead to a higher antigen recognition of EBV-B cells pulsed with PapMV-gp100 compared to peptide-loaded EBV-B cells. We also showed that starvation also led to an increased uptake of PapMV VLP. Hence, the increase cross-presentation of PapMV-gp100 VLP could not solely be explained by an enhanced processing of nanoparticles when autophagy is induced.

In recent years, autophagy has been implicated in several cellular processes [24]. For instance, it has been shown that autophagy is implicated in the regulation of MHC-I internalization and traffic [24, 47]. Loi et al. [47], showed that Atg5- and 7-deficient murine DC have increased surface MHC-I. Contrary to these data, we showed that inhibiting autophagy induction by 3-MA led to a decrease in levels of MHC-I at the surface of EBV-B and T2 cells. These discrepancies could be due to the use of different cellular models and means to inhibit autophagy. Moreover, a non-canonical form of autophagy has been described and is termed LC3-associated phagocytosis (LAP). During LAP, several proteins implicated in the autophagic machinery are recruited to phagosomes such as ATG5, leading to the association of LC3 with the single membrane of the phagosome [48]. By combining both phagocytosis and autophagy LAP couple antigen uptake and degradation. Although this is a matter of a separate study, deciphering the mechanism of entry of PapMV VLP and the implication of LAP in the uptake PapMV VLP would provide a deeper characterization of processes leading to antigen cross-presentation.

The next question to be addressed is how peptides derived from PapMV nanoparticles processing can access MHC-I molecule for loading. Several hypotheses were derived from other models. First, because MHC-I molecules are constantly recycled from the cell surface, endo-lysosomes can fuse with recycling endosomes to allow peptide loading on MHC-I [14, 18, 49]. This hypothesis is supported by the recent work of Makarkov et al. [50], using a plant-derived VLP bearing influenza hemagglutinin (HA). After pulsing of human monocyte-derived macrophages, they detected that a substantial portion of HA-derived peptides were retained in early and/or recycling endosomes where HA colocalized with MHC-I protein [50]. There is also evidence that tubular recycling endosomes can participate in the early stage of autophagosome formation and share their membranes [51]. The second pathway for MHC-I peptide loading is cross-presentation involving exogenous release of newly formed peptides. This form of cross-presentation has been described in immature DC and CpG-activated DC [52]. Autophagy can also be an important regulator of this peptide release. Autophagy has been linked to exosome release from apoptotic/autophagic endothelial cells [53] and multivesicular bodies [54]. These exosomes can fuse with autophagosomes under autophagic conditions [53, 54]. These lines of evidence suggest that autophagy could be implicated in MHC-I peptide loading in the cross-presentation of PapMV VLPs by the vacuolar pathway.

In conclusion, our study provides a first insight in the potential mechanism by which a vaccine platform composed of a chimeric plant VLP is cross-presented. Although further data are required to fully confirm our results, they suggest that PapMV nanoparticles might be processed by a vacuolar cross-presentation pathway implicating the induction of autophagy. A deeper knowledge of this mechanism could help in the design of new vaccine platforms that will have greater autophagy induction properties to promote their cross-presentation by APC and enhance cellular immunity.

## Supporting information

S1 Fig Flow cytometry analysis. **(A)** Gating strategy used to assess PapMV-Alexa Fluor 647 uptake in EBV-B cells. **(B)** EBV-B and T2 cells viability assessed after treatment with inhibitors. Flow cytometry analysis was performed after 3 hours of treatments as this analysis was performed simultaneously with the analysis of PapMV-Alexa Fluor 647 uptake and MHC-I expression. Representative of three indepedent experiments.
(TIF)

S2 Fig Chloroquine and cathepsin S inhibitor treatments do not alter MHC class I surface expression in EBV-B or T2 cells but 3-MA does at high concentrations. EBV-B **(A, C, E)** or T2 cells **(B, D, F)** were treated for 1 hour with chloroquine (ChQ) **(A, B)**, cathepsin S inhibitor (CatS i.) **(C, D)** or 3-methyladenine (3-MA) **(E, F)** at indicated concentrations. Treated cells were labeled with anti-HLA-ABC antibody and MHC-I expression was assessed by flow cytometry. Histograms represent surface MHC-I molecule expression from one representative experiment of three independent experiments. For each experiment, HLA-ABC surface expression was assessed in one technical replicate for each condition. Numbers indicate HLA-ABC MFI from the representative experiment showed. MFI were quantified and normalized relative to the untreated (UT) control. Data are pooled from three independent experiments and are presented as mean ± SEM. Statistical significance of (defined at p <0.05) was calculated using a one-way ANOVA with post-hoc Tukey HSD. ** p <0.01 significantly lower than UT control.
(TIF)

S3 Fig Rapamycin alters antigen presentation in EBV-B and T2 cells. **(A, C)** EBV-B **(A)** and T2 **(C)** cells were pretreated for 1 hour with rapamycin (Rapa) at indicated concentrations and PapMV-gp100 (50 μg/mL) or gp100 peptide (1 μM) was added to pretreated cells without washing. Cells were incubated for 6 hours at 37°C and after extensive washing, were co-cultured with gp100-specific CD8^+^ T lymphocytes for 16–18 hours at a 1:1 ratio. Supernatants were collected and IFN-γ secretion was quantified by ELISA to evaluate MHC-I antigen cross-presentation. Results are presented as mean ± SEM, % of IFN-γ secretion of untreated (UT) cells. Data were pooled from six **(A)** or seven **(C)** independent experiments. For PapMV endocytosis controls, PapMV Alexa Fluor 647 (PapMV-AF647, 10 μg/mL) was added to pretreated EBV-B **(A)** and T2 **(B)** cells and cells were incubated for 3 hours. PapMV-AF647 uptake was assessed by flow cytometry. Histograms represent PapMV-AF647 endocytosis and are representative of three **(A, C)** independent experiments. Numbers indicate PapMV-AF647 MFI from the representative experiment showed. **(B, D)** After rapamycin pretreatment EBV-B **(B)** or T2 **(D)** cells were labeled with anti-HLA-ABC antibody and MHC-I expression was assessed by flow cytometry. Histograms represent surface MHC-I molecule expression from one representative experiment of three independent experiments. Numbers indicate HLA-ABC MFI from the representative experiment showed. MFI were quantified and normalized relative to the UT control. Data are pooled from three independent experiments and are presented as mean ± SEM. Statistical significance (defined at p <0.05) was calculated using one sample t test (**p <0.01) **(A, C)** or a one-way ANOVA with post-hoc Tukey HSD **(B, D)**. When comparing PapMV VLP cross-presentation with the cross-presentation of the control peptide at corresponding inhibitor concentration, a two-tailed unpaired Student’s t-test was performed. Statistical significance was defined at p <0.05 (ns, non-significant).
(TIF)

S4 Fig Starvation alters antigen presentation in EBV-B cells. **(A)** EBV-B cells were incubated in HBSS or pulsed with PapMV-gp100 (50 μg/mL) for 3 hours at 37°C. Cells were collected, protein extracted and resolved by SDS-PAGE using a 15% acrylamide gel, and LC3 was revealed by western blot. The scanned image of the blot was cropped between 20 kDa and 10 kDa. Full-length blot is presented in S5 Fig. Ratio of densitometry of LC3-II/LC3-I bands relative to the untreated (UT) control (n = 1). **(B)** EBV-B were pulsed with PapMV-gp100 (50 μg/mL) or gp100 peptide (1 μM) in complete medium or HBSS for 3 hours. Cells were collected, washed twice, and were subsequently pulsed with PapMV-gp100 (50 μg/mL) or gp100 peptide (1 μM) in complete medium for an additional 3 hours at 37°C. After extensive washing, EBV-B cells were co-cultured with gp100-specific CD8^+^ T lymphocytes for 16–18 hours at a 1:1 ratio. Supernatants were collected and IFN-γ secretion was quantified by ELISA to evaluate MHC-I antigen cross-presentation. Results are presented as mean ± SEM, % of IFN-γ secretion of untreated (UT) cells. Data were pooled from five independent experiments. Statistical significance (defined at p <0.05) was calculated using one sample t test (*p <0.05). When comparing PapMV VLP cross-presentation with the cross-presentation of the control peptide at corresponding inhibitor concentration, a two-tailed unpaired Student’s t-test was performed. Statistical significance was defined at p <0.05 and p values are indicated. **(C)** PapMV Alexa Fluor 647 (PapMV-AF647, 10 μg/mL) was added to EBV-B cells cultured in complete medium or HBSS and cells were incubated for 3 hours. PapMV-AF647 uptake was assessed by flow cytometry. Histograms represent PapMV-AF647 endocytosis and are representative of three independent experiments.
(TIF)

S5 Fig Proposed model of PapMV VLP cross-presentation pathway. ① PapMV VLP are internalized and ② transit through endosomes. ③ Autophagy is induced. ④ Autophagy leads PapMV VLPs in a vacuolar compartment rich in proteases by the fusion with lysosomes with autophagosomes. ⑤ Cathepsin S is implicated in the processing of PapMV VLP which liberates fused epitopes. Other proteases could also participate to PapMV nanoparticle processing. ⑥ Peptide loading of MHC-I molecules could take place in the same vacuolar compartment. ⑦ Peptides are presented on the cell surface of APC. X: PapMV VLP cross-presentation is independent of proteasome activity and TAP. The inhibition of Cathepsin S, lysosome acidification and fusion with autophagosomes (chloroquine and bafilomycin A) as well as the inhibition of autophagy induction (3-methyladenine) alter the cross-presentation of the epitope inserted in PapMV VLPs.
(TIF)

S1 Raw images (PDF)

S1 Data (XLSX)
