# Supplementary figures and images for: MHC class I antigen cross-presentation mediated by PapMV nanoparticles in human antigen-presenting cells is dependent on autophagy
Source: PLoS One. 2021 Dec 31;16(12):e0261987. doi: 10.1371/journal.pone.0261987 (PMC8719699; doi:10.1371/journal.pone.0261987)

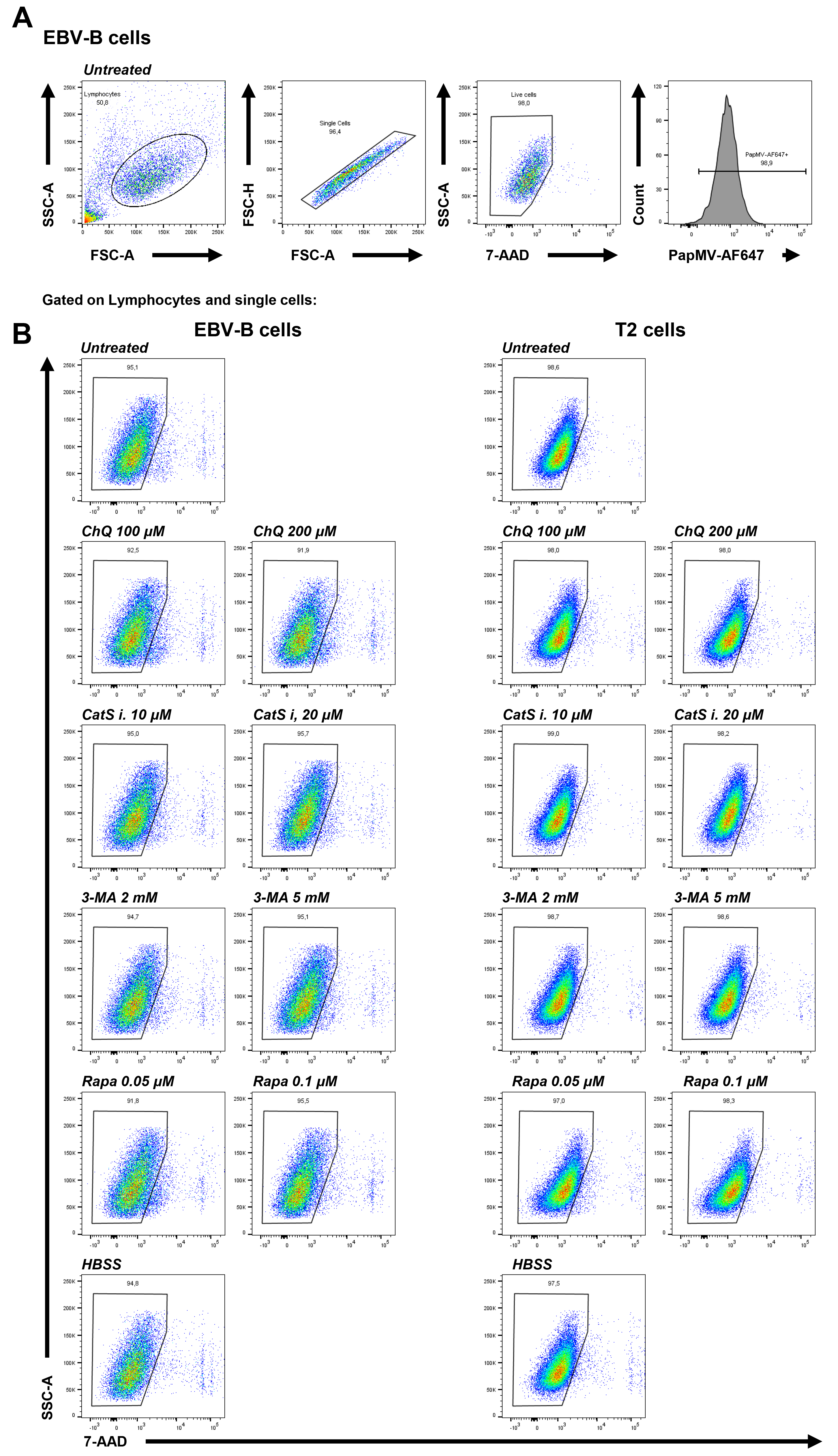

Supplement: S1 Fig — (A) Gating strategy used to assess PapMV-Alexa Fluor 647 uptake in EBV-B cells. (B) EBV-B and T2 cells viability assessed after treatment with inhibitors. Flow cytometry analysis was performed after 3 hours of treatments as this analysis was performed simultaneously with the analysis of PapMV-Alexa Fluor 647 uptake and MHC-I expression. Representative of three indepedent experiments. (TIF) [file pone.0261987.s001.tif]

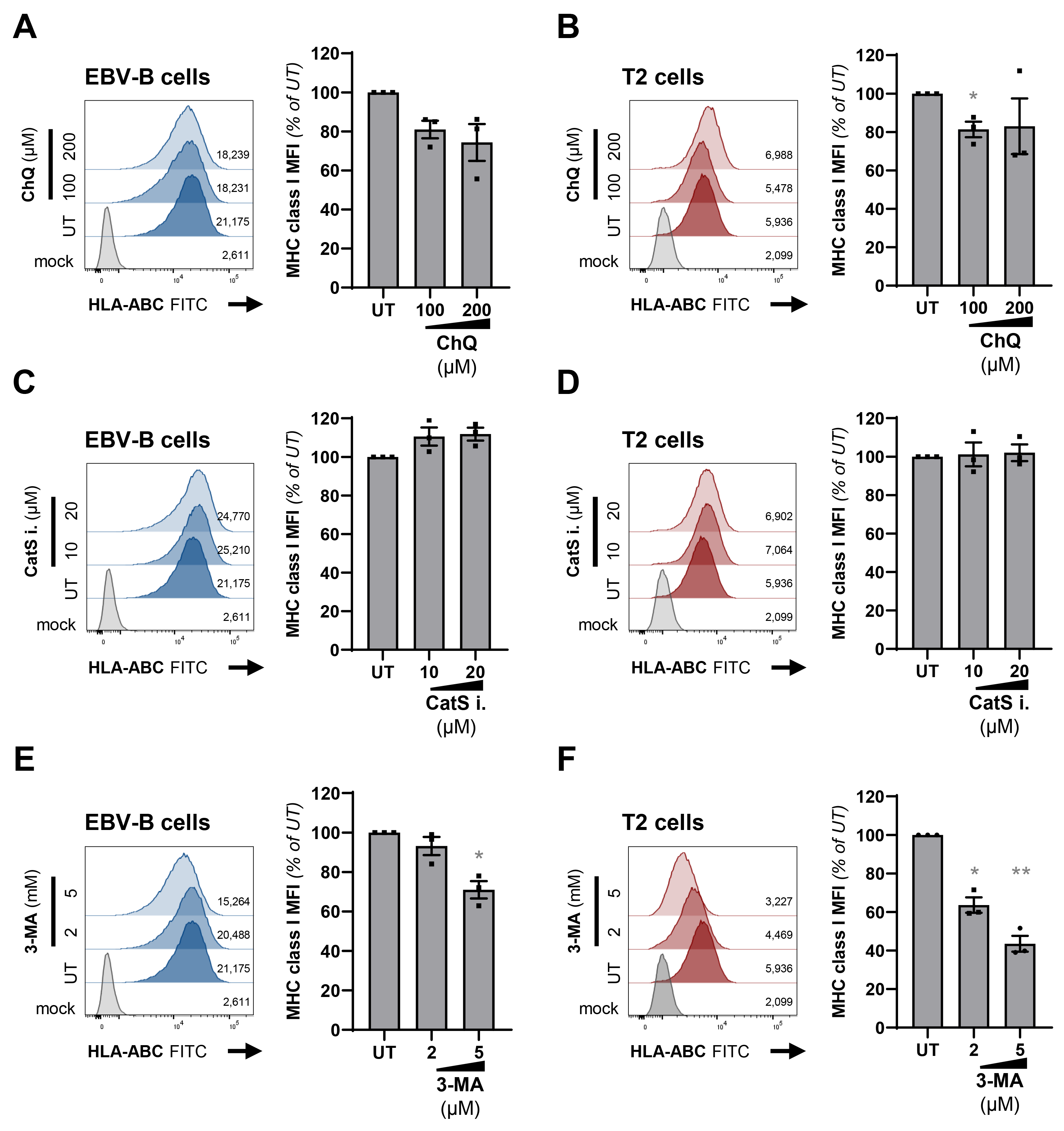

Supplement: S2 Fig — EBV-B (A, C, E) or T2 cells (B, D, F) were treated for 1 hour with chloroquine (ChQ) (A, B), cathepsin S inhibitor (CatS i.) (C, D) or 3-methyladenine (3-MA) (E, F) at indicated concentrations. Treated cells were labeled with anti-HLA-ABC antibody and MHC-I expression was assessed by flow cytometry. Histograms represent surface MHC-I molecule expression from one representative experiment of three independent experiments. For each experiment, HLA-ABC surface expression was assessed in one technical replicate for each condition. Numbers indicate HLA-ABC MFI from the representative experiment showed. MFI were quantified and normalized relative to the untreated (UT) control. Data are pooled from three independent experiments and are presented as mean ± SEM. Statistical significance of (defined at p <0.05) was calculated using a one-way ANOVA with post-hoc Tukey HSD. ** p <0.01 significantly lower than UT control. (TIF) [file pone.0261987.s002.tif]

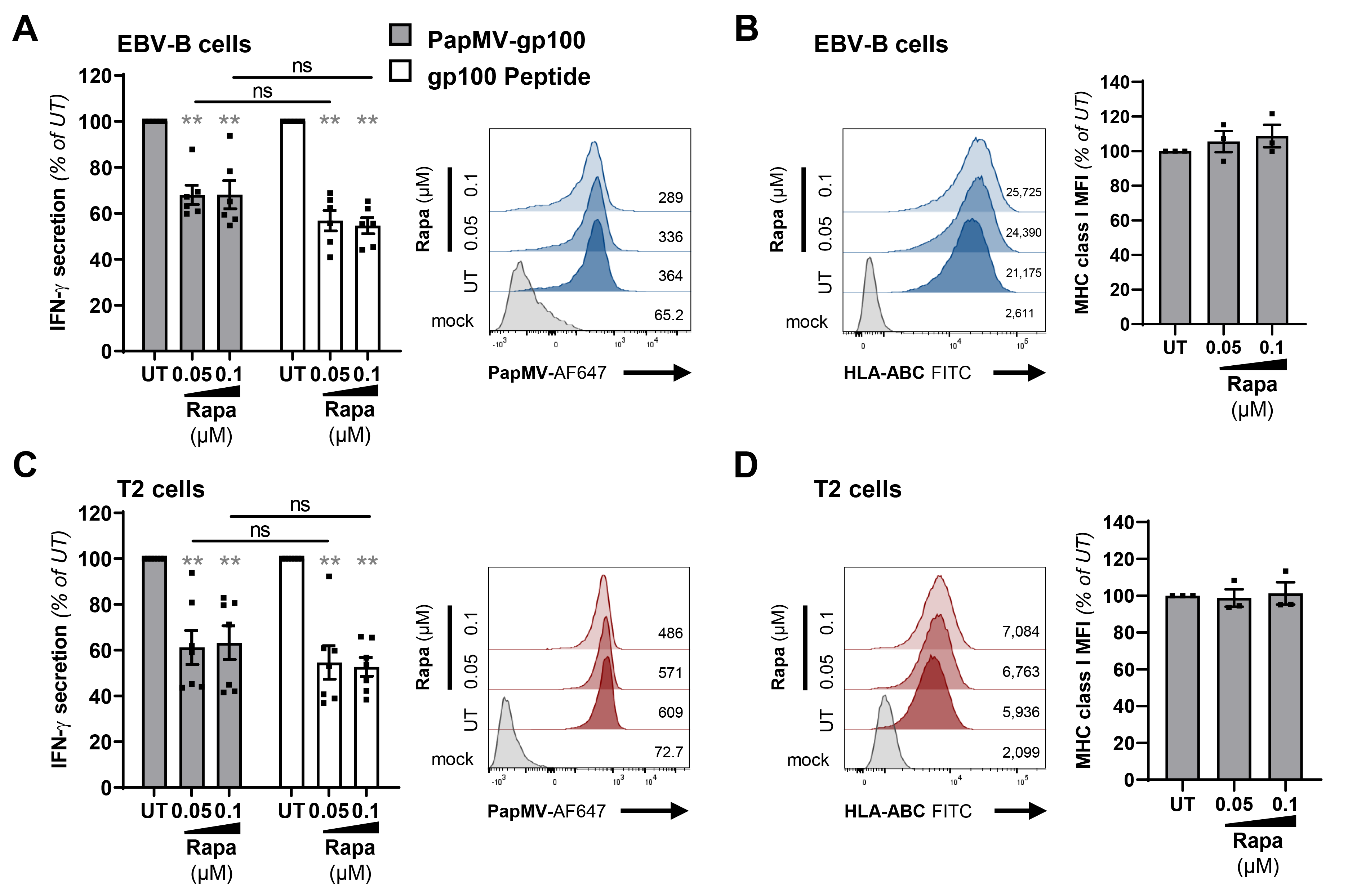

Supplement: S3 Fig — (A, C) EBV-B (A) and T2 (C) cells were pretreated for 1 hour with rapamycin (Rapa) at indicated concentrations and PapMV-gp100 (50 μg/mL) or gp100 peptide (1 μM) was added to pretreated cells without washing. Cells were incubated for 6 hours at 37°C and after extensive washing, were co-cultured with gp100-specific CD8+ T lymphocytes for 16–18 hours at a 1:1 ratio. Supernatants were collected and IFN-γ secretion was quantified by ELISA to evaluate MHC-I antigen cross-presentation. Results are presented as mean ± SEM, % of IFN-γ secretion of untreated (UT) cells. Data were pooled from six (A) or seven (C) independent experiments. For PapMV endocytosis controls, PapMV Alexa Fluor 647 (PapMV-AF647, 10 μg/mL) was added to pretreated EBV-B (A) and T2 (B) cells and cells were incubated for 3 hours. PapMV-AF647 uptake was assessed by flow cytometry. Histograms represent PapMV-AF647 endocytosis and are representative of three (A, C) independent experiments. Numbers indicate PapMV-AF647 MFI from the representative experiment showed. (B, D) After rapamycin pretreatment EBV-B (B) or T2 (D) cells were labeled with anti-HLA-ABC antibody and MHC-I expression was assessed by flow cytometry. Histograms represent surface MHC-I molecule expression from one representative experiment of three independent experiments. Numbers indicate HLA-ABC MFI from the representative experiment showed. MFI were quantified and normalized relative to the UT control. Data are pooled from three independent experiments and are presented as mean ± SEM. Statistical significance (defined at p <0.05) was calculated using one sample t test (**p <0.01) (A, C) or a one-way ANOVA with post-hoc Tukey HSD (B, D). When comparing PapMV VLP cross-presentation with the cross-presentation of the control peptide at corresponding inhibitor concentration, a two-tailed unpaired Student’s t-test was performed. Statistical significance was defined at p <0.05 (ns, non-significant). (TIF) [file pone.0261987.s003.tif]

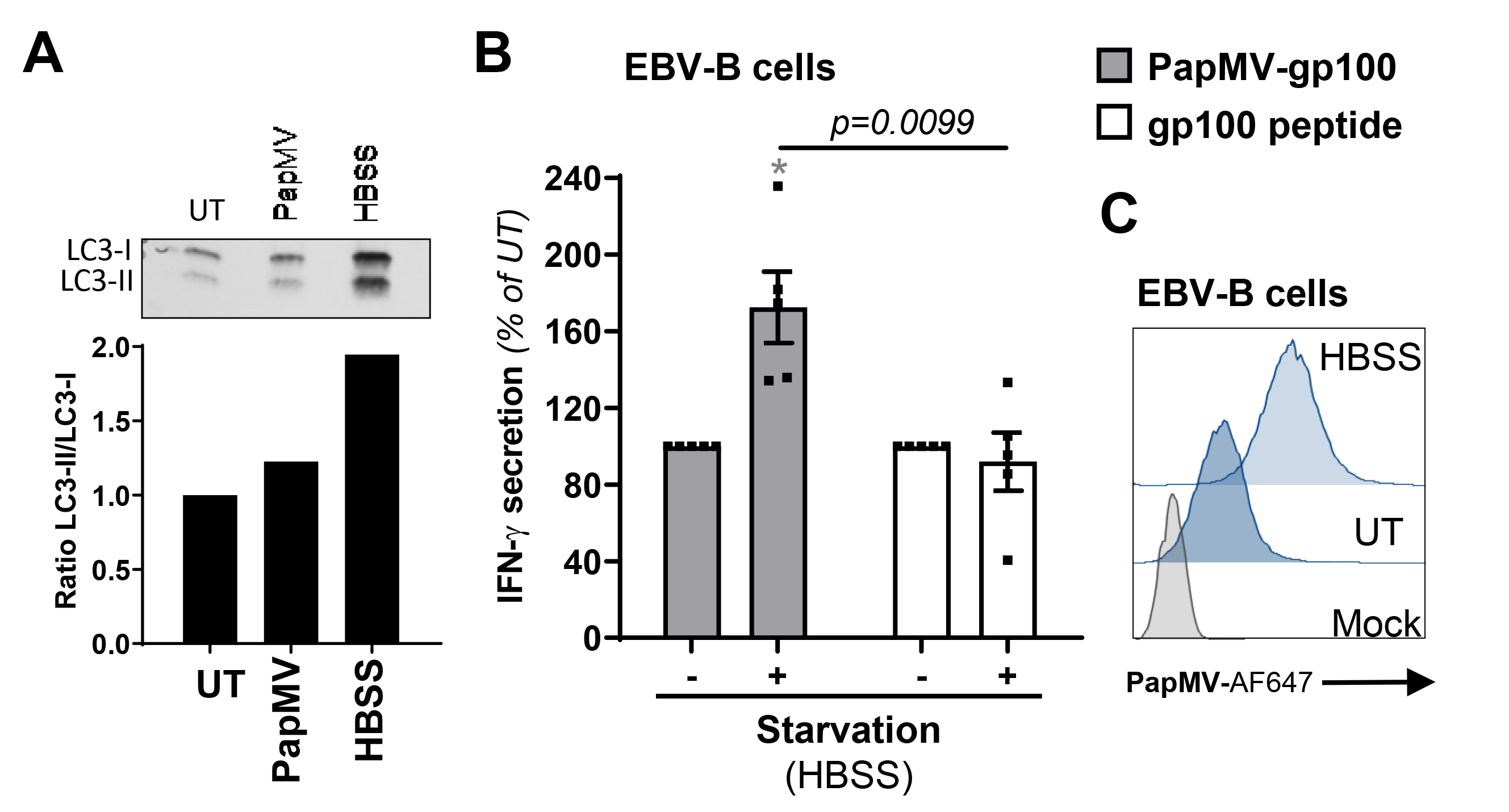

Supplement: S4 Fig — (A) EBV-B cells were incubated in HBSS or pulsed with PapMV-gp100 (50 μg/mL) for 3 hours at 37°C. Cells were collected, protein extracted and resolved by SDS-PAGE using a 15% acrylamide gel, and LC3 was revealed by western blot. The scanned image of the blot was cropped between 20 kDa and 10 kDa. Full-length blot is presented in S5 Fig. Ratio of densitometry of LC3-II/LC3-I bands relative to the untreated (UT) control (n = 1). (B) EBV-B were pulsed with PapMV-gp100 (50 μg/mL) or gp100 peptide (1 μM) in complete medium or HBSS for 3 hours. Cells were collected, washed twice, and were subsequently pulsed with PapMV-gp100 (50 μg/mL) or gp100 peptide (1 μM) in complete medium for an additional 3 hours at 37°C. After extensive washing, EBV-B cells were co-cultured with gp100-specific CD8+ T lymphocytes for 16–18 hours at a 1:1 ratio. Supernatants were collected and IFN-γ secretion was quantified by ELISA to evaluate MHC-I antigen cross-presentation. Results are presented as mean ± SEM, % of IFN-γ secretion of untreated (UT) cells. Data were pooled from five independent experiments. Statistical significance (defined at p <0.05) was calculated using one sample t test (*p <0.05). When comparing PapMV VLP cross-presentation with the cross-presentation of the control peptide at corresponding inhibitor concentration, a two-tailed unpaired Student’s t-test was performed. Statistical significance was defined at p <0.05 and p values are indicated. (C) PapMV Alexa Fluor 647 (PapMV-AF647, 10 μg/mL) was added to EBV-B cells cultured in complete medium or HBSS and cells were incubated for 3 hours. PapMV-AF647 uptake was assessed by flow cytometry. Histograms represent PapMV-AF647 endocytosis and are representative of three independent experiments. (TIF) [file pone.0261987.s004.tif]

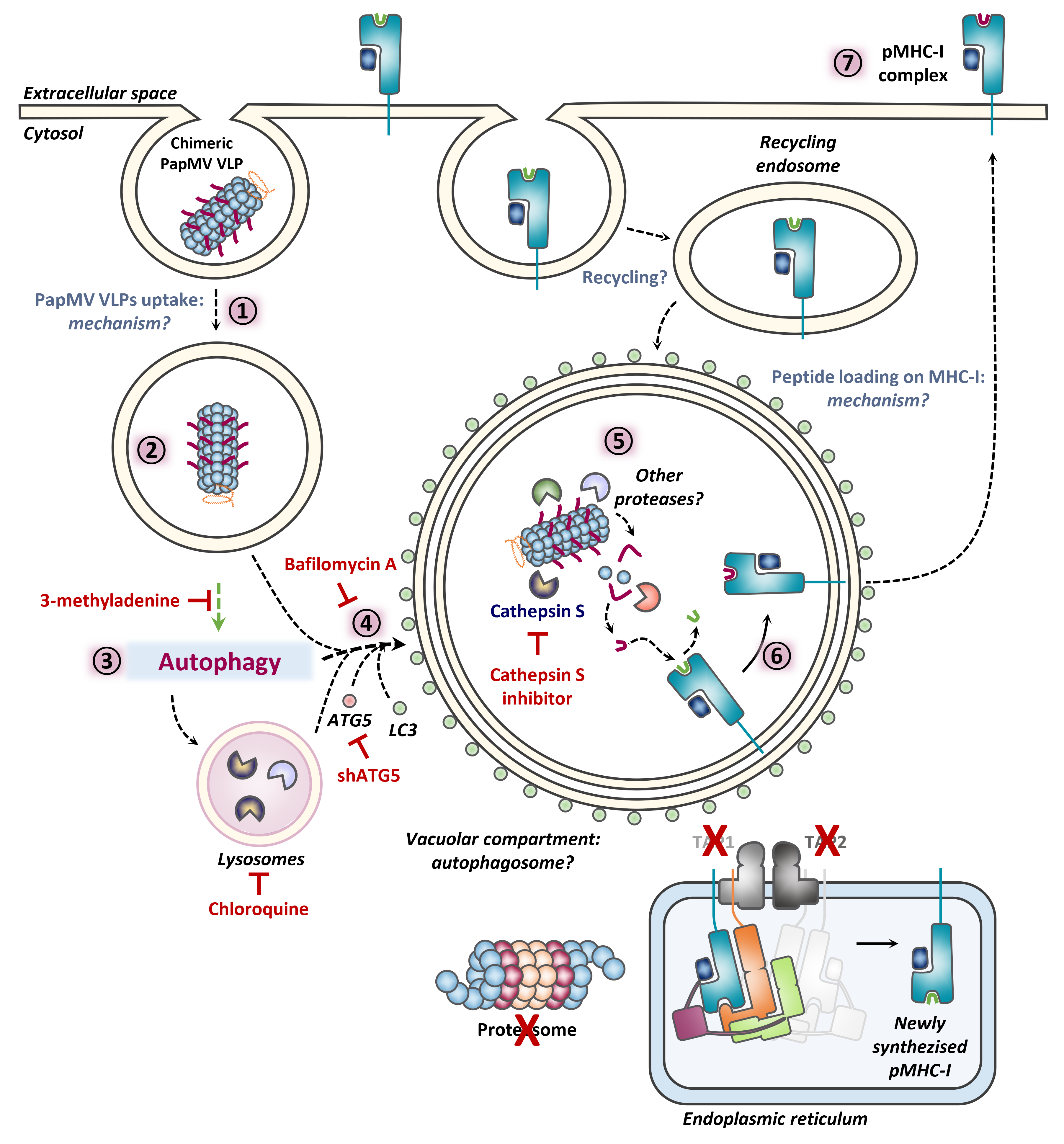

Supplement: S5 Fig — ① PapMV VLP are internalized and ② transit through endosomes. ③ Autophagy is induced. ④ Autophagy leads PapMV VLPs in a vacuolar compartment rich in proteases by the fusion with lysosomes with autophagosomes. ⑤ Cathepsin S is implicated in the processing of PapMV VLP which liberates fused epitopes. Other proteases could also participate to PapMV nanoparticle processing. ⑥ Peptide loading of MHC-I molecules could take place in the same vacuolar compartment. ⑦ Peptides are presented on the cell surface of APC. X: PapMV VLP cross-presentation is independent of proteasome activity and TAP. The inhibition of Cathepsin S, lysosome acidification and fusion with autophagosomes (chloroquine and bafilomycin A) as well as the inhibition of autophagy induction (3-methyladenine) alter the cross-presentation of the epitope inserted in PapMV VLPs. (TIF) [file pone.0261987.s005.tif]
