# Supplementary material for: MHC class I antigen cross-presentation mediated by PapMV nanoparticles in human antigen-presenting cells is dependent on autophagy
Source: PLoS One. 2021 Dec 31;16(12):e0261987. doi: 10.1371/journal.pone.0261987 (PMC8719699; doi:10.1371/journal.pone.0261987)
Supplement: S1 Raw images — (PDF) [file pone.0261987.s006.pdf]

Original blot used in Fig 3A.

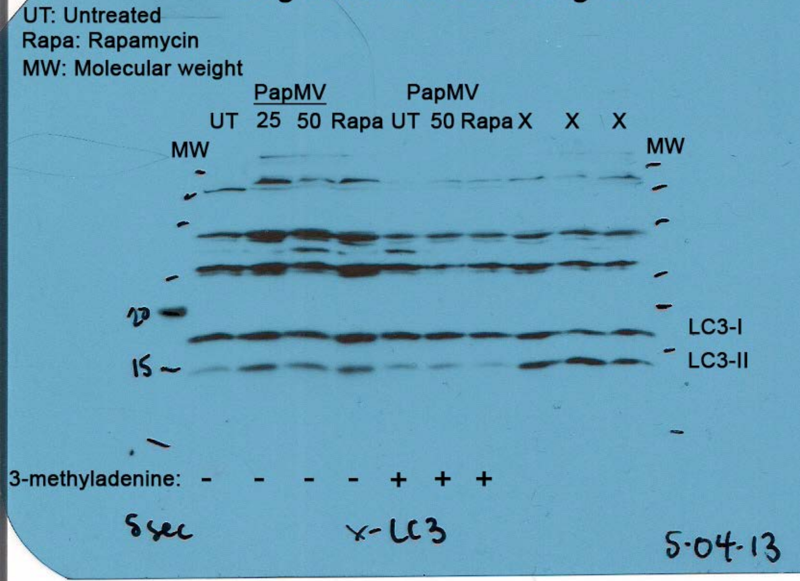

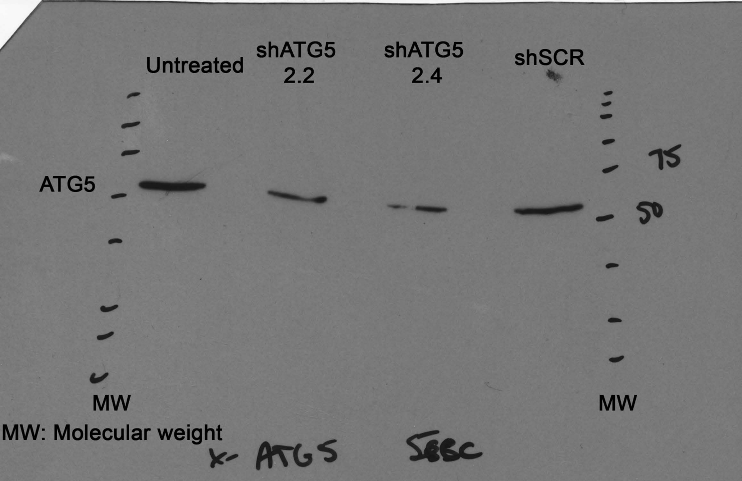

Original blot used in Fig. 4C

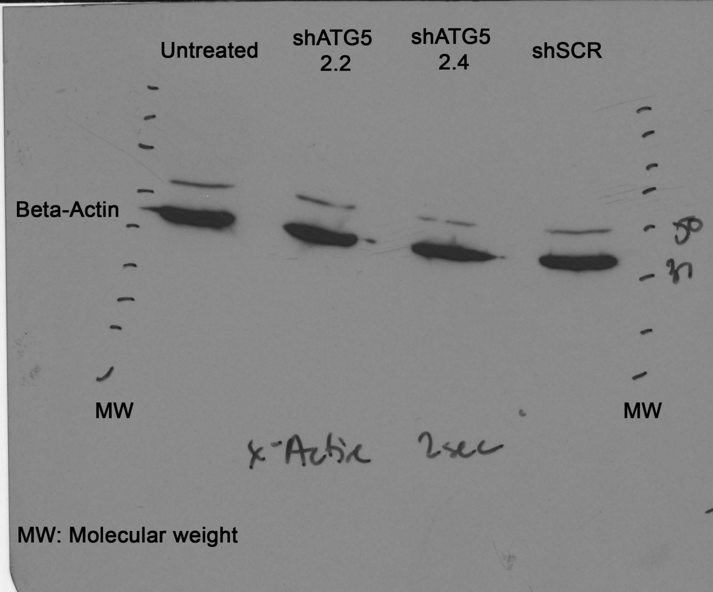

Original blot used in Fig. 4C

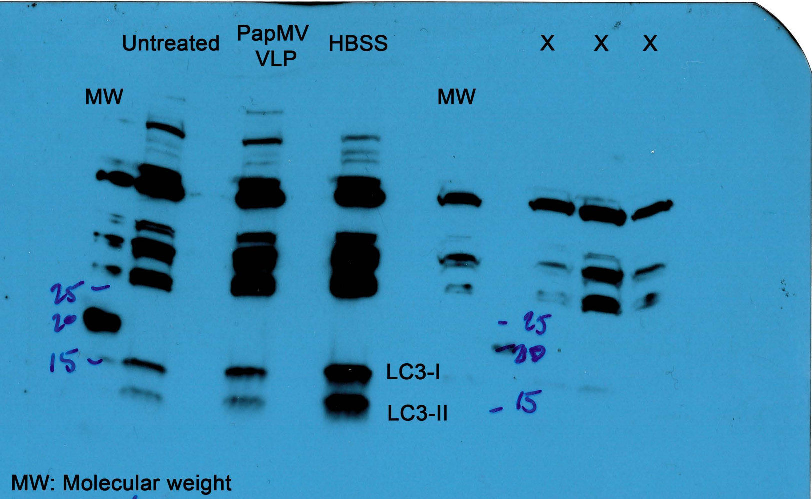

Original blot used in S4A Fig.
